# Deciphering the Mounting Complexity of the p53 Regulatory Network in Correlation to Long Non-Coding RNAs (lncRNAs) in Ovarian Cancer

**DOI:** 10.3390/cells9030527

**Published:** 2020-02-25

**Authors:** Sonali Pal, Manoj Garg, Amit Kumar Pandey

**Affiliations:** 1 Amity Institute of Biotechnology, Amity University Haryana, Panchgaon, Manesar, Haryana 122413, India; sonalipal256@gmail.com; 2 Amity Institute of Molecular Medicine and Stem Cell Research (AIMMSCR), Amity University Uttar Pradesh, Noida 201313, India; mgarg@amity.edu

**Keywords:** ovarian cancer, long non-coding RNAs, p53, effectors, regulators, up-regulated/down-regulated expression, DNA-damage, lncRNAs molecular mechanism, therapeutic implications

## Abstract

Amongst the various gynecological malignancies affecting female health globally, ovarian cancer is one of the predominant and lethal among all. The identification and functional characterization of long non-coding RNAs (lncRNAs) are made possible with the advent of RNA-seq and the advancement of computational logarithm in understanding human disease biology. LncRNAs can interact with deoxyribonucleic acid (DNA), ribonucleic acid (RNA), proteins and their combinations. Moreover, lncRNAs regulate orchestra of diverse functions including chromatin organization and transcriptional and post-transcriptional regulation. LncRNAs have conferred their critical role in key biological processes in human cancer including tumor initiation, proliferation, cell cycle, apoptosis, necroptosis, autophagy, and metastasis. The interwoven function of tumor-suppressor protein p53-linked lncRNAs in the ovarian cancer paradigm is of paramount importance. Several lncRNAs operate as p53 regulators or effectors and modulates a diverse array of functions either by participating in various signaling cascades or via interaction with different proteins. This review highlights the recent progress made in the identification of p53 associated lncRNAs while elucidating their molecular mechanisms behind the altered expression in ovarian cancer tumorigenesis. Moreover, the development of novel clinical and therapeutic strategies for targeting lncRNAs in human cancers harbors great promise.

## 1. Background

Ovarian cancer (OC) is the most prevalent and fatal gynecological malignancy affecting a multitude of females worldwide. According to the global statistics data, OC is the 7th most common cancer and 8th most common cause of mortality from cancer in females across the globe. The Globocan study predicts that by 2035 there will be a worldwide increase in the incidence of OC to 3, 71,000 and an increase in deaths to 254,000. China and India have the largest number of patients diagnosed with OC followed by the USA [1].

Early diagnosis and resistance to clinical treatment are the greatest challenges due to the lack of uniformity in the pathogenesis of OC. Generally, ovarian tumors can develop in any of the three cell types: epithelial cells, sex cord-stromal cells (including granulosa, theca, hilus cells), and germ cells (oocytes) [2]. A large number of studies have shown that 90% of malignant OCs are epithelial in origin while remaining are non-epithelial in origin and are less invasive [3,4,5]. Epithelial ovarian cancer (EOC) is of two types mucinous and non-mucinous. It is further classified based on histopathology, molecular genetic analysis, and immunohistochemistry into serous carcinoma, endometrioid carcinoma and clear cell carcinoma account for 70%, 10%, and 10%, respectively [6]. Serous carcinoma is of two subtypes: high-grade serous carcinoma (HGSC), the most common and aggressive form of OC, while low-grade serous carcinoma (LGSC) is relatively uncommon (<5%) [7]. The available tumor staging protocols which define the extent of tumor spread are the American Joint Committee on Cancer (AJCC) Tumor, Node, Metastasis (TNM) system and International Federation of Gynecology and Obstetrics (FIGO) staging system [2]. The current modalities available for the detection of ovarian cancer, at early-stages, are pelvic examination and transvaginal sonography (TVS). Moreover, there are two noninvasive tests that are available in the clinics for detecting OCs are cancer antigen-125(CA125) and human epididymis secretory protein E4(HE-4). These are U.S. Food and Drug Administration (FDA)-approved biomarkers for early diagnosis of OC. Besides, assembled panels of biomarkers are available called ‘composite biomarkers’ which are not yet widely available [8].

## 2. Outline of TP53 and LncRNA

HGSC accounts for 70% of all OC and is one of the rapidly growing human malignancies. It mostly originates in the fallopian tube epithelium and has a poor 5-year survival rate of 9–34%. The *tumor suppressor 53 (TP53)* gene is one of the well-characterized tumor suppressor genes and it has been shown to be crucial for cellular homeostasis. A high frequency of *TP53* gene mutations has been observed in HGSC [9]. The *TP53* gene in human tumors often undergoes missense mutations [10] and these mutations have been shown to drive the initiation, progression, and development of several human tumor types. The *TP53* mutations are widely distributed in all coding exons of the *TP53* gene, mostly concentrated in the DNA binding domain particularly in exons 4–9. About 30% of all mutations in this domain have six “hotspot” residues (residues R175, G245, R248, R249, R273, and R282) [11]. The 3′-untranslated region(3′UTR) and non-coding part of the *TP53* gene is susceptible to both somatic and germline mutations [12]. The *TP53* tumor suppressor is the guardian of the genome [13]. Dysregulation in the TP53 pathway is thought to be the foundation leading to tumorigeneses. Conventionally, mouse double minute2 (MDM2) which is a ubiquitin ligase induces p53 and degrades it via the ubiquitin proteasomal pathway. The p53 is a homotetramer protein induced in effect to diverse stress signals like hyperproliferative signals, hypoxia, ribonucleotide depletion, oxidative stress. Most importantly, during the DNA-damage, phosphorylation of p53 occurs at multiple sites catalyzed by kinases which disrupt the association of the MDM2-p53 complex, leading to stabilization of p53 protein [14]. This suggests that p53 is regulated at both translational [15] and transcriptional levels [16]. It is a DNA binding transcription factor that regulates the expression of a plethora of genes [17]. Some of the major target genes that are regulated by p53 encode proteins which are crucial in the preservation of genome integrity, differentiation, cellular proliferation, promoting apoptotic cell death, cell cycle arrest and senescence [18,19]. HGSC harbors *TP53* mutations in 96% of the cases [20,21]. Characterization of HGSC for *TP53* mutation and assessment of TP53 expression levels are made possible with the help of massive-parallel sequencing and immunohistochemistry [22]. The International Agency for Research on Cancer (IARC) database leads to the identification of 2329 of *TP53* mutations in human OC (http://www-p53.iarc.fr/), out of which 70.33% are missense mutations, while others are point mutations [23].

The Encyclopedia of DNA Elements (ENCODE) project determined that the human genome encodes 25,000 protein-coding genes, representing 1.5% of the total genome sequence. The 60–70% portion of the human genome encompasses non-protein-coding sequences like non-coding RNAs (ncRNAs), regulatory sequences and introns [24,25]. It is quite interesting to note that some of the ncRNAs specifically the lncRNAs have been revealed as bonafide p53 transcriptional targets [26]. Based on the transcript size, ncRNA falls under two classes: small ncRNA (18 to 200 nts) and long ncRNAs (200 nts to >100 kb in size). With the dawn of the functional annotation of the mammalian genome (FANTOM) and ENCODE transcript mapping projects, which lead to the identification and characterization of lncRNAs. The lncRNAs are the novel, independent, functional and an indispensable class of noncoding RNAs transcripts that do not encode proteins. Like mRNA, their transcription is regulated by RNA polymerase II, a 5′ cap is present with many exons and polyadenylated. The lncRNAs may be non-polyadenylated, derive from pol III promoters. Previously, lncRNAs have been considered as transcriptional noise in the genome [27]. The expression levels of lncRNAs are well regulated than that of the protein-coding genes. The lncRNAs comprises of significant domains, such as RNA, DNA and protein-binding domains that perform the various biological functions [28].

## 3. LncRNAs Modes of Action

LncRNAs exhibits varied modes of action, such as signaling lncRNAs, act as a molecular signal and can function as markers in significant biological events. Molecular decoy lncRNAs come into play by imitating and competing with their consensus DNA-binding motifs. These sponge-binding protein factors such as transcription factors and chromatin modifiers that direct broad changes in the cell’s transcriptome like GAS5, Lethe and PANDA. Guide lncRNAs are coupled from transcriptional co-regulators or chromatin regulatory protein complexes and bind them to specific genomic regions to regulate transcription like Kcnq1ot1andlincRNA-p21. Scaffold lncRNAs; include HOTAIR, XIST, and NRON. These lncRNAs possess distinct domains that bind with various protein factors and may altogether bring specific regulatory proteins in proximity with one another to function as a complex and impact transcriptional activation or repression. Enhancer lncRNAs, this loop the chromosome with the help of a looping mechanism, to bring various target proteins together [29]. Nevertheless, being ubiquitous, the functional aspect of nuclear lncRNAs is gauged widely including their involvement in gene regulation at both transcriptional and post-translational levels as well as post-transcriptional modifications of proteins [30]. lncRNAs participates in both transcriptional repression and activation [31]. However, at the post-transcriptional level, lncRNAs intervenes in mRNA organization, stability, translation and affects protein-protein interactions. In addition to this, LncRNAs are involved in dosage compensation and genomic imprinting [32].

Based on the genomic proximity with the neighboring annotated genes, lncRNAs are classified as: (1) Sense lncRNAs, transcribed in the same direction as the protein-coding genes with at least one exon overlapped. For example, Gas5 (growth arrest-specific 5) and MALAT1 (metastasis-associated lung adenocarcinoma transcript 1) belong to this category [33]. (2) Antisense lncRNAs, also called natural antisense transcripts (NATs), is the second most abundant, overlapping one or more exons of another transcript on the same or opposite strand and overlap with sense mRNAs at the 5′ (divergent NAT or head to head) or 3′-ends (convergent NAT or tail to tail). For example, HOTAIR transcribed in an antisense manner from the mammalian homeobox transcription factors C(HOXC) locus on chromosome 12q13.13 [34]. (3) Bidirectional lncRNAs that reside within the proximity of the promoter or enhancer region in a divergent direction. These can generate enhancer-associated RNAs(eRNAs) [35] and promoter-associated long RNAs (PALRs) within a few hundred bp [36]. (4) Intronic lncRNAs, derived from an intron of a coding gene without overlapping exons at either end. For example, COLD AIR, located in the first intron of the flowering repressor locus (FLC) [37]; (5) Intergenic lncRNAs, also known as large intervening ncRNAs, lincRNAs, or stand-alone lncRNAs. It is the largest subclass of lncRNAs in humans. These exist as an independent unit between the two protein-coding genes, at least 5 kb from both sides. For example, H19, Xist, and lincRNA-p21; (6) Pseudogene lncRNAs, are the product of reverse-transcribed mRNA inserted into the genome. For example, *PTENP1* [29] With the assistance of large-scale MiTranscriptome bioinformatics tools, estimates have revealed that there are nearly 60,000 lncRNAs and approximately 21,000 protein-coding genes. Recent studies unveiled that a strong correlation exists between lncRNAs and cancer [27]. Moreover, nearly 8000 lncRNAs genes were found to be associated with varieties of cancer types, including 514 lncRNAs in OC.

## 4. The Alliance of LncRNAs with TP53 Pathway in Ovarian Cancer

The strong functional association of lncRNAs with TP53 is quite intriguing in the realm of holistic gene regulatory network and such association has been unveiled in numbers of models both in vitro and in vivo [38]. For the identification of the TP53-responsive lncRNAs, various high throughput techniques are being used including lncRNAs-specific microarray, RNA-seq, genome-wide chromatin Immunoprecipitation (ChIP-seq). Also, mouse model systems serve to be an effective medium in such recognition [39]. TP53 regulates some of the lncRNAs while some dictate the expression or the activity of p53.Both the mechanisms operate in the sink either to maintain p53 proteins at the finest level or to implement a downstream function in response to p53 activation.

## 5. LncRNAs as Effectors in the p53 Network: LncRNAs Regulated by p53

**Figure 1:**
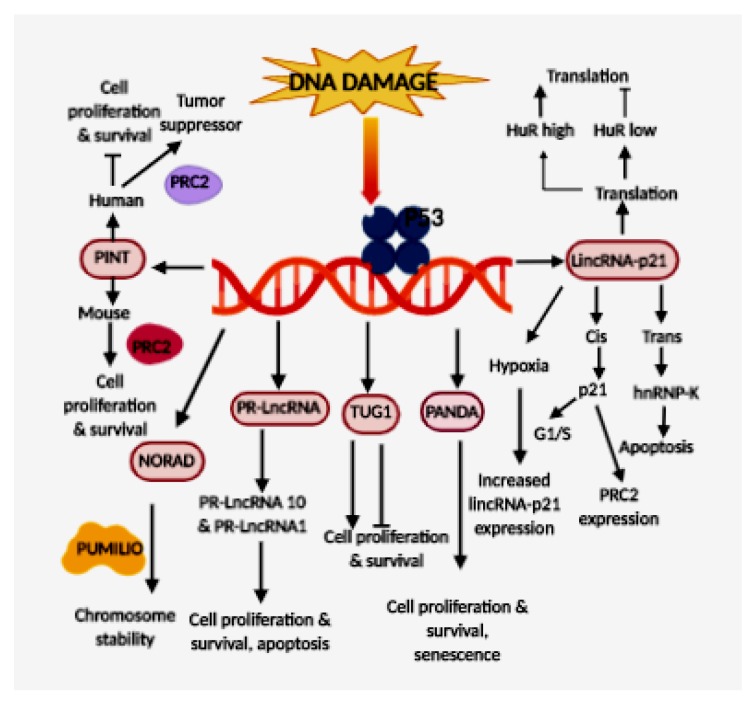
The role of p53-effectors of lncRNAs that are regulated by p53; LincRNA-p21 functions in both cis and trans. In cis, it regulates the expression of its neighboring gene p21 and in trans, it interacts with RNA binding protein hnRNP-K to exert its proapoptotic role. The lincRNA-p21 expression is induced during hypoxia. LincRNA-p21 regulates translation in the cytoplasm; PANDA has a pro-survival function as it promotes cell survival and inhibits apoptosis; Human PINT interacts with PRC2 and acts as a tumor-suppressor, whereas mouse ortholog increases cell survival and proliferation; PR-lncRNA1 and PR-lncRNA10 have proapoptotic function and they inhibit cell survival and proliferation; TUG1 promote or inhibit cell proliferation and cell cycle progression depending on the tumor type; NORAD interacts with PUMILIO proteins to maintain genomic stability.

Several lncRNAs could be activated or inhibited by TP53. For example, lncRNAs in mouse LincRNA-p21[Trp53cor1] and PINTA, B, C-[lncpint] and lncRNAs in Humans PANDA [PANDAR], lncRNAs [LINC-PINT], PR-lncRNA-1, NORAD[LINC00657], TUG1, PVT1, eRNAs, LINP1 [RP11-554I8.2] and DDSR1 (Figure 1). These are capable of modulating gene expression downstream of TP53.

### 5.1. LincRNA-p21 [Long Intergenic Non-Coding RNA p21]

The lincRNA-p21 was identified by tiling microarrays performed from p53 activation following DNA damage in mouse embryonic fibroblasts (MEFs) and lung cancer cells derived from mice express KRAS.LincRNA-p21 is a first broadly studied p53-induced lncRNA well characterized as a crucial regulator of the p53 network [26]. LincRNA-p21 is named corresponding to its location lying approximately 15 kb upstream of the *CDKN1A* gene (coding for p21 protein). Also known as tumor protein p53 pathway corepressor 1 (Trp53cor1), mapped to human chromosome 6 and chromosome 17 of the mouse [40]. LincRNA-p21 has been displayed to be expressed at eight molecules per cell and has a short half-life of about less than 2 h [41]. LincRNA-p21 has an impact on the p53 network by primarily functioning in cis to turn on the expression of the neighboring gene, *CDKN1A (p21)* [41]. LincRNA-p21 exerts its function by interacting and recruiting hnRNP-K (heterogeneous nuclear ribonucleoprotein K). It is a chromatin-associated factor that targets the specific gene promoters and represses their transcription that leads to p53-dependent apoptosis [42]. The knockdown of lincRNA-p21 resulted in the altered expression of a large number of target genes that are usually repressed by p53. This clearly indicates that lincRNA-p21 serves as one of the repressors in the p53 pathway [43]. The murine LincRNA-p21 is restricted to the nucleus and is down-regulated in multiple tumors [44]. On the contrary, human lincRNA-p21 is located in the cytosol and performs various functions. For instance, it suppresses mRNA translation by interacting with the RNA-binding protein HuR. It is a critical player in hypoxia-enhanced glycolysis [45]. The p53-regulated lincRNA-p21 prevents somatic cell reprogramming [46].

Linc-Mkln1 (LINC-PINT) resides in the nucleus and is a direct transcriptional target of p53 [47]. The murine LINC-PINT shares several similarities with its human ortholog, such as both are transcriptionally regulated by p53. LncRNAs acts as a tumor suppressor and is downregulated in multiple human cancers including lung, breast, and uterine corpus endometrial [48]. The LINC-PINT function is highly dependent on a conserved sequence CE1 which binds to polycomb repressive complex 2 PRC2 that results in the silencing of genes involved in the process of cell invasion. Moreover, LINC-PINT not only inhibits the ability of cells to invade in vitro but also reduces the engraftment potential of the cells in vivo, in colorectal (HCT116) and lung (A549) adenocarcinoma cells [49].

### 5.2. Taurine Upregulated Gene 1 (TUG1)

TUG1 is highly conserved lncRNA and originally recognized to play a significant role in the process of retina differentiation in the mouse. TUG1 is 7.1 kb lncRNA and transcribed from human chromosome 22q12.2 [50]. RNA immunoprecipitation (RIP-ChIP) experiments revealed that TUG1 performs its repressor role via molecular scaffold by recruiting the chromatin-modifying complexes PRC2 [51]. Moreover, it has been found to be associated with repressive nuclear foci/PcG bodies present on the promoter that interacts with a specific protein called polycomb 2 protein (Pc2), a component of the PRC1 [52]. TUG1 is ubiquitously expressed and plays an important role in various cancer types [53]. TUG1 has been demonstrated to be upregulated in ovarian cancer [54]. It inhibits apoptosis and promotes cell proliferation by up-regulating the expression of aurora kinase A [55]. ChIP and luciferase assays have demonstrated that TUG1 is a direct transcriptional target of p53. TUG1 promoter contains conserved binding sites for p53. It binds to the putative response element present in the promoter region of TUG1.TUG1 expression is induced by p53, binds to PRC2 and suppresses genes involved in cell-cycle regulation. Thus, TUG1 may serve as a downstream transcriptional repressor in the p53 pathway to repress the progression of the cell cycle in response to DNA damage. The TUG1 is only induced in p53-wild type when compared to p53-mutant cells [51]. It is assumed that TUG1 may be closely linked with p53 and human malignancies. However, further research is required to verify this assumption.

### 5.3. p21-Associated Noncoding RNA DNA Damage-Activated (PANDAR)

It is a mono exonic lncRNA activated in response to DNA damage and oncogene-induced senescence [56] in a p53-dependent manner and transcribed in the reverse direction of p21 [57]. It is located 5 kb upstream of the transcription start site (TSS) of the *CDKN1A* gene promoter. The functional aspect of PANDAR characterized with the aid of high-resolution tiling array revealed its important role in cell cycle progression, cellular senescence, and apoptosis [58]. PANDAR shares a significant dual-role with A-subunit of nuclear transcription factor Y (NF-YA), a trimeric unit associated with tumor progression [59]. Firstly, TP53 binds to p21 TSS and regulates the activity of both *p21 [CDKN1A]* and PANDAR in response to DNA damage. This results in the induction of transcription of both PANDAR and p21 in the nucleus. As a result, PANDAR fuses with NF-YA and shuts down the transcription of pro-apoptotic genes such as *APAF1, BIK*, and *FAS* by inhibiting NF-YA from binding to its promoter [60]. Further findings suggest that PANDAR is a TP53 downstream effector. In OC, higher expression of PANDAR has been displayed to play a crucial role in the chemoresistance mechanism [61]. For instance, during DNA damage, PANDAR is found to inhibit apoptosis in human fibroblasts. Secondly, PANDAR has been shown to promote cell survival in non-small cell lung carcinoma (NSCLC) in which low expression level of PANDAR permits NF-YA to up-regulate Bcl2 expression, an anti-apoptotic gene [62]. PANDAR expression significantly increased after treatment with a drug that causes DNA damage like doxorubicin [57] and etoposide. Furthermore, PANDAR participates in the stabilization of p53 protein in the NF-YA-independent manner and regulates it post-translationally [60].

Some of the lncRNAs that not distinctly categorized and are regulated by p53 are LOC285194, LOC401317, and LNC_BC060912. The Loc285194 is a lncRNA, located on chromosome 3q13.3. It is a p53-regulated tumor suppressor functioning as a molecular sponge for *miR-211* [63]. It promotes cell growth [64] and is downregulated in multiple tumors [65]. In primary osteosarcoma samples, chromosome location 3q13.3 is subjected to high copy number alterations (CNAs) and loss of heterozygosity (LOH) [66]. Ectopic expression of loc285194 suppresses tumor cell growth both in vitro and in vivo. For instance, tumor growth and migration are reported in colon cancer with an upregulated level of miR*-211*, whereas miR-211 activity repressed upon overexpression of LOC285194 [67]. Interestingly, the LOC401317 functions to block the cell cycle progression and induces apoptosis in tumor cells. Therefore, it is crucial to elucidate its role as a tumor suppressor [68]. On the contrary, p53 stimulation suppresses the LNC_BC060912 lncRNA expression, which is directly or indirectly controlled by p53. The LNC_BC060912 has been displayed to inhibit apoptosis and is involved in DNA damage repair [69]. However, LNC_BC060912 mechanism of action remains unexplored.

### 5.4. Plasmacytoma Variant Translocation 1 (PVT1)

PVT1 is homologous evolutionary conserved lncRNA first identified in murine plasmacytomas, while in humans it resides in 8q24 region near to the *c-Myc* locus [70]. It is another p53-regulated lncRNA transcribed from a noncoding locus *(PVT1)* that comprises a cluster of six microRNAs. In gastric cancer cells, *PVT1* acts as an oncogene which represses few tumor suppressor genes, like p15 and p16 and known to promote cell proliferation.

PVT1 is involved in G1 arrest and also interacts with PRC2 and its core member EZH2 and represses p15 and p16 [70,71]. Interestingly, the binding of p53 to the first intron of the PVT1 induces lncRNAs and miR-1204 expression. miR-1204 is a miRNA member of the cluster located in the PVT1 locus [72]. The overexpression of the PVT1 in breast and OC results in the regulation of the apoptotic pathway thereby conferring cisplatin resistance [73]. On the contrary, PVT1 interacts with both p53 and tissue inhibitor of matrix metalloproteinase (TIMP), to achieve the antineoplastic action of carboplatin and docetaxel [74].

### 5.5. Damage Induced Noncoding (DINO)

DINO is a 951 bp, conserved lncRNA that acts as a tumor suppressor [75]. It is located near to the p53- binding site and upstream of the *CDKN1A (P21)* promoter. In response to DNA damage, DINO participates in the regulation of cell cycle arrest and p53 signaling. DINO deletion in vivo results in reduced sensitivity to radiation. The DINO-p53 interaction with the C-terminal RNA binding region of the TP53 controls many activities such as maintenance of p53 stability in both humans and MEFs cells as well as regulates p53 signaling during DNA damage. Also, few p53 target genes like *DDB2; GADD45A* and *RRM2* are induced via DINO. To comprehend the role of DINO, studies were performed on knockout mouse models in which inactivation of DINO promoter resulted in deregulated response to doxorubicin which revealed that mouse DINO acts in a trans manner, similar to human DINO [76].

## 6. LncRNAs that Dictate the Expression of p53: p53 Regulators of LncRNAs

**Figure 2:**
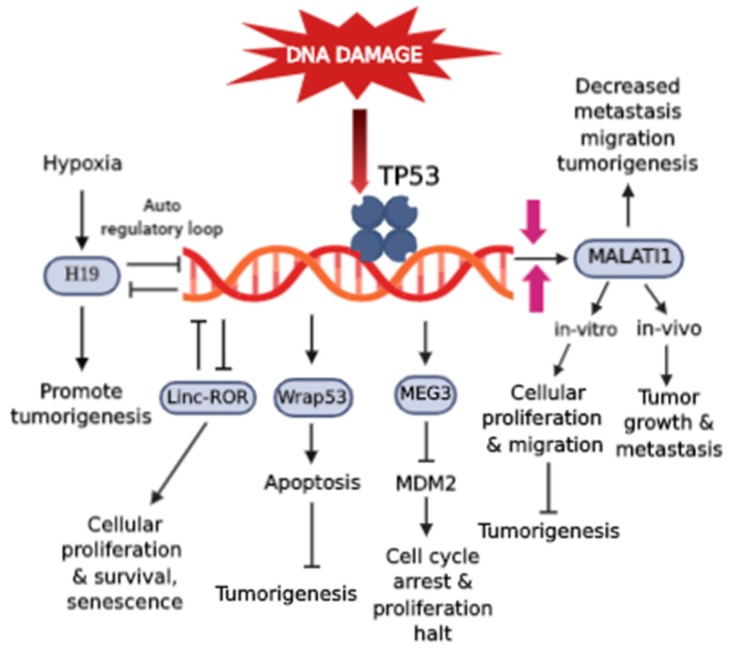
The role of p53-regulators of lncRNAs that directly and/or indirectly regulate p53 activity either repressing and/or activating its expression levels. MEG3 downregulates MDM2 expression that activates p53 resulting in cell cycle arrest and inhibits cell proliferation and tumorigenesis. Other lncRNAs like H19, RoR, act in an autoregulatory loop in cooperation with TP53.

At the post-transcriptional level, several lncRNAs either directly or indirectly regulate p53 activity either restraining or activating its expression levels. Some of the Human lncRNAs are MALAT1/(NEAT2), MEG3, H19, Wrap53, LincRNA-RoR (Figure 2.)

### 6.1. Metastasis-Associated Lung Adenocarcinoma Transcript1 (MALAT1)

MALAT1 (also known as NEAT2) acts as a repressor of p53 [77] originally identified in lung cancer [78]. It is highly abundant, nuclear lncRNA overexpressed in varieties of tumors including breast and colon. It is involved in alternative splicing and metastasis [25]. MALAT1 increases cell proliferation in vitro and promotes tumor formation in nude mice. Evidence of oncogenic activity of MALAT1, in the MALAT1 knockout model which unveiled that MALAT1 regulates the expression of metastasis-related genes which leads to metastasis of early-stage non-small cell lung cancer and is an efficient prognostic marker for the patient with poor survival rates [77]. The depletion of the MALAT1 initiates double-stranded DNA damage response in human fibroblasts which results in the activation of TP53 and other downstream target genes. This leads to G1 cell cycle arrest. MALAT1 controls the cell cycle progression via interaction with pro-proliferative genes such as *E2F1* [79] and decreased the *E2F1* level as a result of TP53 activation [80]. MALAT1 RNA level significantly upregulated in cells infected with DNA oncoviruses highlighting regulation of MALAT1 expression by p53 [81]. In OVCAR3, knockdown of MALAT1expression inhibits cell proliferation, migration and invasion leading to G0/G1 cell cycle arrest and apoptosis. On the other hand induction of MALAT1 with transforming growth factor β1 (TGF-β1) results in cell proliferation, migration of OC cells [82].

### 6.2. Maternally Expressed Gene 3 (MEG3)

MEG3 functions as a tumor suppressor and is downregulated in several human malignancies [83]. Several knockout mouse models are used to study the function of MEG3 in vivo [25]. MEG3 functions in p53-dependent as well as p53-independent manner [84]. In the absence of p53 MEG3 is found to inhibit cell proliferation [85]. Importantly, MEG3 upregulates the expression of p53 target gene growth differentiation factor 15 *(*GDF15*)* in the presence of p53 strongly suggests that *GDF15* is a MEG3 target gene [84,86]. A multifaceted expression of ectopic MEG3 is examined in breast cancer cells [87]. Moreover, certain anti-cancer drugs such as etoposide [88], genistein [89] and resveratrol can induce *GDF15* expression by activation of p53 [90]. In OC, *MEG3* induces p53 as ectopic expression of *MEG3* suppresses proliferation and growth to induce apoptosis [91].

### 6.3. WD Repeat-Containing, Antisense to p53 (Wrap53)

Wrap53 is a natural antisense transcript of p53 [92]. It is located upstream of the p53 and acknowledged to inhibit p53 expression at the post-transcriptional level [93]. The isoform of Wrap53 contains a complementary sequence to the first exon of TP53 and is capable of regulating p53 expression while the other two isoforms β and γ fails to do so. The functional aspect ofWrap53 in correlation to p53 is quite interesting. The knockdown of Wrap53 during DNA damage terminates TP53 induction. On the contrary, the ectopic expression of Wrap53 induces p53-dependent apoptosis [92]. Wrap53 and p53 mRNAs form RNA-RNA duplex, which stabilizes p53 [94]. Interestingly, a recent study has revealed that Wrap53 regulates p53 levels via physically interacting with highly conserved zinc finger protein (CTCF) CCCTC- binding factor [95]. The binding of the TP53 gene promoter to CTCF helps in the maintenance of an open chromatin configuration which prevents epigenetic silencing [96].

### 6.4. Long Intergenic Non-Protein Coding RNA-Regulator of Reprogramming (LINC-ROR)

Linc-ROR is a 28-base sequence located at 18q21.31 having four exons, first identified in induced pluripotent stem cells (iPSCs) [97,98]. Studies revealed that it is down-regulated in numerous cancer types [99] including hepatocellular cancer (HCC) [100], endometrial cancer (EC) [101], breast cancer (BC) [102], pancreatic cancer (PC) [103], and nasopharyngeal carcinoma (NPC) [104]. Some of the regulatory roles played by linc-ROR are the maintenance of stem cell pluripotency, triggering the epithelial-mesenchymal transition (EMT) [105], hypoxia, in the proliferation of cancerous cells acting both as promotor/inhibitor and in self-renewal of glioma stem cells (GSCs) via inhibition of KLF4 expression. ROR and p53 are functionally interdependent [106]. ROR directly interacts with heterogeneous nuclear ribonucleoprotein I (hnRNP I) also known as PTBP1 present in the cytoplasm to repress p53 translation [107]. p53 activates ROR expression by binding with the p53-response element (p53RE) in the ROR promoter. Moreover, is known to inhibit p53-mediated cell cycle arrest and apoptosis [106]. p53 expressions are controlled by the autoregulatory feedback loop between p53 and ROR. Linc-ROR unites with p53, NRF2, and c-Myc forming a cluster of proteins to regulate the expression in response to DNA damage [106]. In OC cells, linc-ROR induces epithelial-mesenchymal transition. It also serves as an important molecule in the invasion and metastasis in OC cells [108]. In nasopharyngeal carcinoma, linc-ROR suppress the p53 signal pathway due to which NPC resists chemotherapy [104]. The high cellular stress results in the induction of nuclear factor-erythroid 2-related factor (NRF2) [109] which provides defense against chemical carcinogenesis [110].

### 6.5. H19

H19 is ~2.7 kb long and maternally expressed lncRNA [111]. Functionally, p53 and H19 oppose each other, and their expression is tightly regulated. The p53 induces DNA demethylation [112] of the imprinting control region (ICR) at the upstream to the H19 gene; therefore, negatively regulating the expression of H19 by suppressing its promoter activity [113]. Under hypoxic stress conditions, wild-type p53 suppresses the elevation of H19 RNA in the nucleus [114]. Studies conducted on genome-wide methylation profiling demonstrated that methylation changes at numerous gene loci and H19lncRNA participates in the process of gene methylation via regulating S-adenosylhomocysteine hydrolase (SAHH). SAHH is a mammalian enzyme capable of hydrolyzing S-adenosylhomo- cysteine (SAH) and has the ability to methylate different cellular components such as DNA, RNA, and proteins [115].

In OC tumor tissues, H19 has been observed to induce cell cycle arrest and apoptosis [116]. Upregulated expression of H19 results in HGSC recurrence [117] and drug resistance toward cisplatin chemotherapy [118]. LOH and LOI in H19 gene has been displayed to be involved in advanced clinical stages of OC and carcinogenesis [119]. Furthermore, studies reported in other cancers such as gastric and bladder cancer signify that p53 and H19 share strong functional crosstalk [120]. Many reports suggest that the oncogenic activity of H19 is dependent on miRNAs. For example, H19 acts as a molecular ‘sponge’ for the let-7 family of miRNAs. The Let-7 is a well-characterized tumor suppressor miRNA. The Let-7 functions to suppress the target gene expression. It binds to imperfect complementary sequences in the mRNA, resulting in translational repression and mRNA degradation [121]. In OC, the metastasis and tumor suppressor miR-675 targets H19 and their expression being negatively correlated. H19 acts as an oncogene by upregulating miR-675. These regulate the proliferation and invasion of cells by regulating extracellular signal-regulated kinases1/2(ERK) and EMT. Moreover, miR-675 is one of the important miRNAs that participate in migration, invasion and tumor cell proliferation in OC [122]. The summary of p53 linked lncRNA in ovarian cancer is presented in Table 1.

## 7. p53-Induced LncRNA that Directs DNA Repair

p53 maintains genome stability by interacting with the *Mlh1*, which is necessary for the management of the DNA repair process and p53-mediated tumor suppression [125]. DNA is very prone to damage caused due to various factors. DNA double-strand breaks (DSBs) are a hallmark in cancer that results in chromosomal rearrangements and mutations. The two different mechanisms for repair of the double-stranded break (DSBs) are homologous recombination (HR) and non-homologous end-joining (NHEJ) [9]. The lncRNAs are indispensable players in guarding the integrity of the genome by participating in DSB repair pathways. Recently, it is revealed that during the DNA damage, PURPL [9] and PINCR, the p53-induced lncRNAs [126] directly regulates p53 or interacts with p53 target genes subsets such as *BTG2*, *RRM2B*, and *GPX1* that functions in the arrest of cell cycle and apoptosis. Some of the lncRNAs, such as *DDSR1-*DNA damage sensitiveRNA1, damage-induced lncRNAs (dilncRNAs), NORAD, Antisense lncRNAs, LINCP1 and GUARDIN, participates in the process of DNA repair [127].

### 7.1. DNA Damage Sensitive RNA1 (DDSR1)

DDSR1 is activated upon DNA damage with various DNA-damaging agents like camptothecin, etoposide, and neocarzinostatin regulated by the ATM NF-kβ pathway [128]. DDSR1 negatively regulates p53 gene expressions. The knockdown of *DDSR1* significantly decreases cell proliferation in U2OS, PC3, and A549 cell lines [129]. DDSR1 recruits HR factors like *BRCA1*, heterogeneous nuclear ribonucleoprotein U-like 1 (hnRNPUL1) and RAP80 to the double-strand breaks and repairs the DNA via homologous recombination participates in DNA repair pathways [127].

### 7.2. NORAD

NORAD is a highly abundant, conserved, cytoplasmic lncRNA and is activated after doxorubicin treatment and is indirectly regulated by p53 [130]. NORAD and its downstream regulator PUM1/2 proteins functions in the maintenance of genome stability while the absence of NORAD results in aneuploidy. NORAD interacts with cellular protein PUMILIO, thereby preventing the inhibition of target genes that are involved in DNA replication, mitosis, and DNA repair by PUMILIO [131].

### 7.3. Antisense LncRNAs

These play direct roles in DSB repair. The majority of lncRNAs sequences interact via RNA-mediated DNA repair mainly observed in retroviruses, telomere synthesis [132]. Anti-sense lncRNAs are transcribed from both undamaged and damaged locus and couples at the site of double-stranded DNA break [DSB] with homologous DNA. Anti-sense lncRNA regulation is possible via both *cis/trans* mechanisms, but cis is preferred. In *cis,* lncRNA facilitates end joining by forming R-loops modulated via HR whereas; in trans lncRNA interacts with chromatin at the site of DSB and can form both DRBP and R-loops [133].

### 7.4. LncRNA in Non-homologous End-Joining Pathway1 (LINP1)

LINP1 is overexpressed in colon tumor cell lines and metastatic TNBC and regulates p53 indirectly [134]. LINP1participates in the NHEJ DNA repair pathway in association with two proteins involved in this process named Ku80 and DNA-PKcs [135]. The knockdown of LINP1 resulted in IR-induced DNA damage. *LINP1* binds to miR-29 whose regulation is directly controlled by p53 suggesting that miR-29 may be a mediator of p53- regulated LINP1expression.

### 7.5. GUARDIN

GUARDIN is transcriptionally controlled by p53. GUARDIN, in association with p53, promotes cell survival and maintains genome stability and in vitro decreased GUARDIN level elicits senescence growth arrest and increased apoptosis [136]. GUARDIN expression increases significantly in response to DNA damage (DDR) as reported in lung, breast adenocarcinoma, osteosarcoma, in untransformed fibroblasts and colorectal cancer cells [133]. GUARDIN has the potential to act as a competitive endogenous RNA (ceRNA). MicroRNA, miR-34a transcribes GUARDIN from its promoter region and is a well-known target of p53 [137]. With the help of RNA, pull-down followed mass spectrometry [138], which revealed that GUARDIN interacts with the tumor suppressor gene *BRCA1* that initiates DNA repair damaged nuclear loci and supports *BRCA1–BARD1* interaction by acting as RNA scaffold [139]. *BRCA1*-associated ring domain1 *(BARD1)* is a ribonucleoprotein (RNP) complex formed by *BRCA1* [140].

## 8. Regulation of p53-Mediated Subsets of Genes Via LncRNAs

The p53 induced lncRNAs can regulate the subsets of p53 targets genes in a sequence-specific manner [141] in two different ways: (i) by direct or indirect up-regulation of genes and, (ii) by indirect repression of genes. The most widely accepted mechanism of action is the association of lncRNAs with chromatin that can act as an activator or repressor in altering the gene expressions of a subset of genes. The specificity of lncRNAs for binding to the particular gene locus is very crucial in the regulatory process, and various molecular mechanism has evolved to associate the chromatin at a definite locus (A) by directly interacting with DNA forming R-loops, RNA-DNA hybrid (B) Indirect association of DNA via a DRBP, DNA- and RNA-binding protein (C) by binding to pre-mRNA and (D) by binding to pre-mRNA via an RBP, RNA-binding protein [142]. The regulatory regions of p53 targets may be present in enhancers, promoters, and insulators that interact with lncRNAs. These increase the expression of some p53 target genes by facilitating chromatin looping or may repress or induce gene expression via histone modifications at regulatory sites [143].

## 9. Methodologies Targeting LncRNAs for Therapeutic Applications

The lncRNAs are promising and potentially ideal targets in clinical implications. To date, various strategies devised that efficient target lncRNAs which include siRNAs, antisense oligos, ribozymes, CRISPR, ZNFs and TALENs, using small molecules, nanobodies, aptamers, and RNA decoys. Some of the different methodologies stated are as follows: (1) Blocking/silencing lncRNAs mechanism of action via (a) RNA interference (b) CRISPR/Cas9 (c) Antisense oligonucleotides and (2) functional inhibition (a) Small molecules inhibitors [144].

### 9.1. The RNA Interference (RNAi)

This is a direct and influential approach that functions by silencing the lncRNAs [145,146,147]. The process of degradation of targeted lncRNA accompanies the use of small interfering RNAs (siRNAs), short hairpin RNAs (shRNAs), and miRNAs delivered into the cells where the antisense strands are with RNA-induced silencing complex (RISC). This approach is highly tissue-specific and provides temporally-controlled gene expression [143]. For instance, as a result of siRNA/shRNA-mediated silencing of MALAT1 which subsequently leads to a decrease in tumor progression, cell motility, and viability along with apoptosis in adenocarcinoma, cervical and HEPG2 cell [148]. Moreover, the silencing of H19 and HULC with this method can potentially result in the reduced growth of tumors in xenografts and is capable of altering the expression of myriad genes [149]. The usage of this approach is extended to preclinical models and to multiple cancer types such as in breast, hepatocellular and pancreatic cancers which includes siRNA-mediated downregulation of HOTAIR [150] downregulation of PCA3 instate cancer [151]. In xenograft models, the available alternative methods to knockdown lncRNAs in vivo are Dicer- and Argonaute- dependent RNA silencing [150], and the use of liposome/nanoparticle-delivered siRNAs that have potential to inhibit tumorigenesis and metastasis [152]. It includes dioleoyl phosphatidylcholine (DOPC)-based nanoliposomes that have been designed to deliver nucleotide based-therapeutics like siRNA, miRNA, lncRNA, and ASOs for in vivo and clinical use [153]. RNAi is a broad and highly efficient approach, but it withholds certain limitations such as the necessity of suitable delivery vehicles for proper cellular uptakes like liposomes [154], nanoparticles [155] or viruses to preventing their degradation and accumulation.

### 9.2. CRISPR/Cas9 Genome Editing System

Clustered regulatory interspaced short palindromic repeat (CRISPR/Cas-based) approach efficiently target lncRNAs in cancerous cells [156]. The method consists of a guide RNA to drive the Cas9 endonuclease, which cleaves a specific sequence in the target lncRNAs. Other genome editing tools, such as zinc-finger nucleases (ZFNs), transcription activator-like effector nucleases (TALENs) are also available [157]. For example, detection of MALAT1 by the ZFN or TALEN method in lung cancer cells and human breast cancer cells knockout of lncRNAs such as UCA1, lncRNA-21A, and AK023948 by the CRISPR/Cas9 system [158]. The development of non-viral delivery methods for CRISPR-Cas9 genome editing is quite appealing for its use in therapeutics. However, the major limitation is the complexity in spatial localization of lncRNAs that can even localize to the nucleus rendering the technique to be less effective

### 9.3. Antisense Oligonucleotides (ASOs)

This approach uses a modified synthetic oligonucleotide including ASO gapmers, duplex RNA, and locked nucleic acids [159,160]. Locked nucleic acids (LNAs) are delivered into the cells, which later enters into the nucleus and binds to their complementary endogenous RNA targets via base-pairing [151]. The RNA–DNA duplex triggers RNase-H-dependent cleavage. Various cancer models have been tested that uses ASOs approach to knockdown lncRNAs that results in inhibition of tumor growth and progression in vivo. For instance, in human lung cancer cells, ASO-mediated knockdown of MALAT1 prevents primary tumor metastasis [161]. LNAs targetPVT1 in sensitizing OC cells to cisplatin [162] and mimic GAS5 to induce apoptosis [163]. Although this approach efficiently targets the lncRNAs in vivo, the major disadvantage is the cytotoxicity and poor cellular uptake.

### 9.4. Small Molecule Inhibitors

High-throughput screening has provided a wider platform for identification of small inhibitor molecules both in vivo and in vitro that block lncRNAs-protein interactions [163,164,165], such as HOTAIR-PRC2, ANRIL-CBX7, PCAT-1-PRC2, and H19-EZH2 [151]. For instance, the interaction between HOTAIR with either PRC2 or LSD1is blocked by two small molecule inhibitors (2-PCPA and DZNep) that decrease the metastatic activity of the breast cancer cells [166]. The use of multiple small-molecule inhibitors, for instance, in OC, the upregulated expression of EZH2 serves as an effective therapeutic target which is now in clinical trials [167].

## 10. Concluding Remarks

The multifaceted function of p53 with its prominent tumor suppressor activity and involvement of lncRNAs in this scenario is quite intriguing. It has paved the way to dig more into the gigantic and unstoppable p53 gene regulatory network. Interestingly, the abundance and heterogeneity in lncRNAs with a varied mechanism of action made them an essential mediator of p53. A recent finding on the functional aspect of lncRNAs indicates that the deregulated expression of lncRNAs is associated with tumorigenesis in multiple tumor types. At present several lncRNAs are coordinated by p53 transcription factor but are also required by p53 to express its tumor suppressor activity. In OC, very few p53-linked lncRNAs are discovered that regulate a plethora of regulatory circuitries including their association in tumorigenesis initiation & progression, metastasis, apoptosis and some of them are even known to participate in DDR. Given the complexity of the network, lncRNAs act both as an oncogene as well as a tumor suppressor and their linkup with p53 as effectors and regulators further broadens the platform for identification and characterization of greater numbers of p53 linked lncRNAs in OC.

Moreover, an animal model may be a useful tool for the functional analysis of lncRNAs. Overcoming the limitations in terms of therapeutic strategies that target lncRNAs is quite challenging, such as insufficient cellular uptake and cytotoxicity of the ASOs as well as lack of an efficient delivery system for RNAi whereas small molecule inhibitors avoid these shortcomings. Therefore, targeting lncRNAs can be of prime importance in human malignancies. The functional versatility and the diverse range of mechanisms qualify lncRNAs as a promising and potential target in future diagnostics and clinical implications.

## Figures and Tables

**Table 1:** Elucidating the role of various p53 linked lncRNAs in ovarian cancer.

p53 –linked lncRNA in Ovarian Cancer	Upregulated	Down Regulated	Aberrant Phenotype	Reference
H19	up	down	Promote proliferation, metastasis, EMT and inhibits apoptosis. Recurrence marker	[113,114,115,116,117,118,119]
MALAT1	up	-	Promote proliferation, migration, and invasion. Metastasis and inhibits apoptosis. Recurrence marker	[77,78,79,80,81,82]
TUG1	-	-	Promote proliferation, metastasis and inhibits apoptosis	[54,55]
PVT1	up	-	Acts as an oncogene. Promote proliferation, migration, invasion and cisplatin resistance	[73,74]
MEG3	-	down	Inhibits apoptosis, autophagy and promotes proliferation, cisplatin- resistance	[88,89,90,91,123]
PANDA	-	down	Cisplatin-resistance	[61]
Wrap53	-	-	Repairs double stranded DNA breaks	[124]
LINC-ROR	up	-	Promote cell proliferation, migration and invasion in vitro	[108]

## Abbreviations

OC: ovarian cancer; EOC: Epithelial ovarian cancer; LncRNAs: Long non-coding RNAs; HGSOC: Serous ovarian cancer; BRCA1: Breast cancer-associated gene 1; MDM2: Mouse double minute2; GENCODE: Encyclopedia of DNA Elements; (ENCODE): comprehensive expression analysis of the Encyclopedia of DNA Elements; miRNAs: MicroRNA; *NF-YA*: Nuclear transcription factor Y subunit alpha; ceRNAs: Competing endogenous RNAs.
